# Supplementary material for: Targeted protein degradation in mycobacteria uncovers antibacterial effects and potentiates antibiotic efficacy
Source: Nat Commun. 2024 May 14;15:4065. doi: 10.1038/s41467-024-48506-8 (PMC11094019; doi:10.1038/s41467-024-48506-8)
Supplement: Supplementary file 1 — Supplementary Information [file 41467_2024_48506_MOESM1_ESM.pdf]

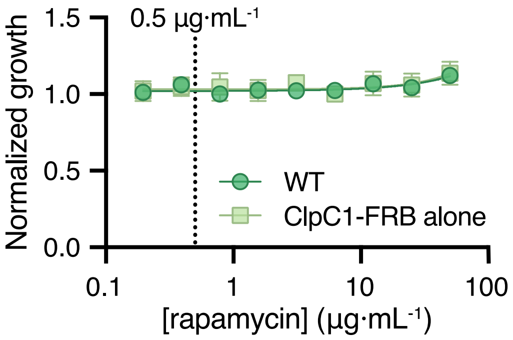

Supplementary Figure 1 | Rapamycin does not restrict mycobacterial growth at the concentrations used in this work.
Half-maximal minimum inhibitory concentration (MIC_{50}) dose response measuring the sensitivity of *Msm* strains to rapamycin. Primary concentration of rapamycin used in this work indicated by dotted line. Data are mean ± s.d. of three technical replicates and are representative of two independent experiments. Related to Fig. 1. Source data are provided with this paper.

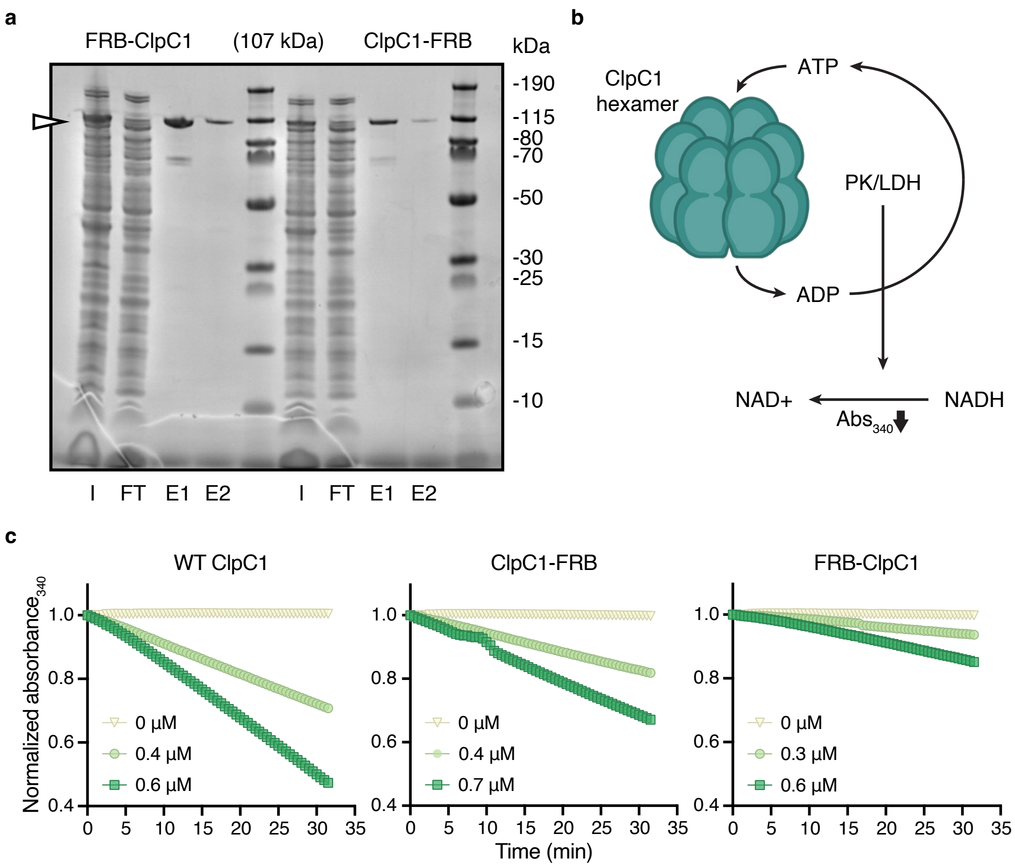

Supplementary Figure 2 | C-terminally tagged *Mtb*ClpC1-FRB retains ATPase activity *in vitro*.

a SDS-PAGE of purified tagged *Mtb*ClpC1 proteins expressed in BL21 cells. Arrow denotes expected size of fusion proteins. I = input; FT = flow-through; E1 = elution 1, 100 mM imidazole; E2 = 200 mM imidazole. **b** Schematic of ATP/NADH coupled *in vitro* assay to measure ClpC1 ATPase activity. PK = p yruvate kinase. LDH = lactate dehydrogenase. **c** Absorbance at 340nm measuring *in vitro* ATPase activity of WT ClpC1 (left), ClpC1-FRB (middle), or FR B-ClpC1 (left) with the indicated concentrations of each protein. For **c**, data are individually plotted measurements, normalized to time = 0 h, and are repr esentative of two independent experiments. **b** Created with BioRender.com, released under a Creative Commons Attribution-NonCommercial-NoDerivs 4.0 International license Related to Fig. 1. Source data are provided with this paper.

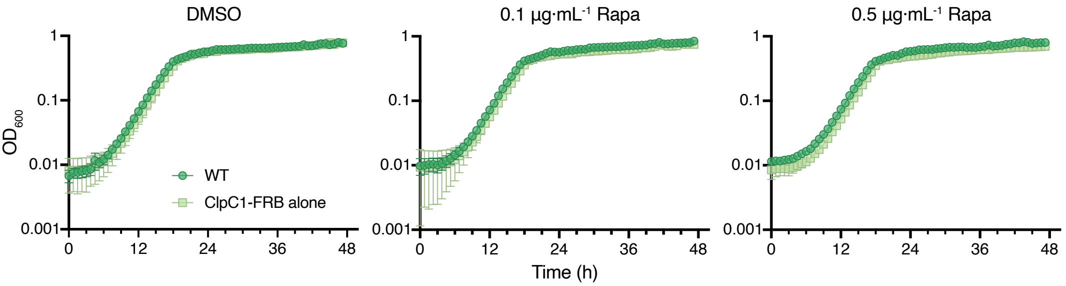

Supplementary Figure 3 | The ClpC1-FRB strain is not growth-impaired compared to WT *Msm*.
Optical density of bacterial cultures at 600nm measuring the growth kinetics of the indicated *Msm* strains over time when supplemented with DMSO (left), 0.1 µg·mL^{-1} rapamycin (middle), or 0.5 µg·mL^{-1} rapamycin (left) with shaking at 37°C. Data are mean ± s.d. of three technical replicates and are representative of two independent experiments. Related to Fig. 1. Source data are provided with this paper.

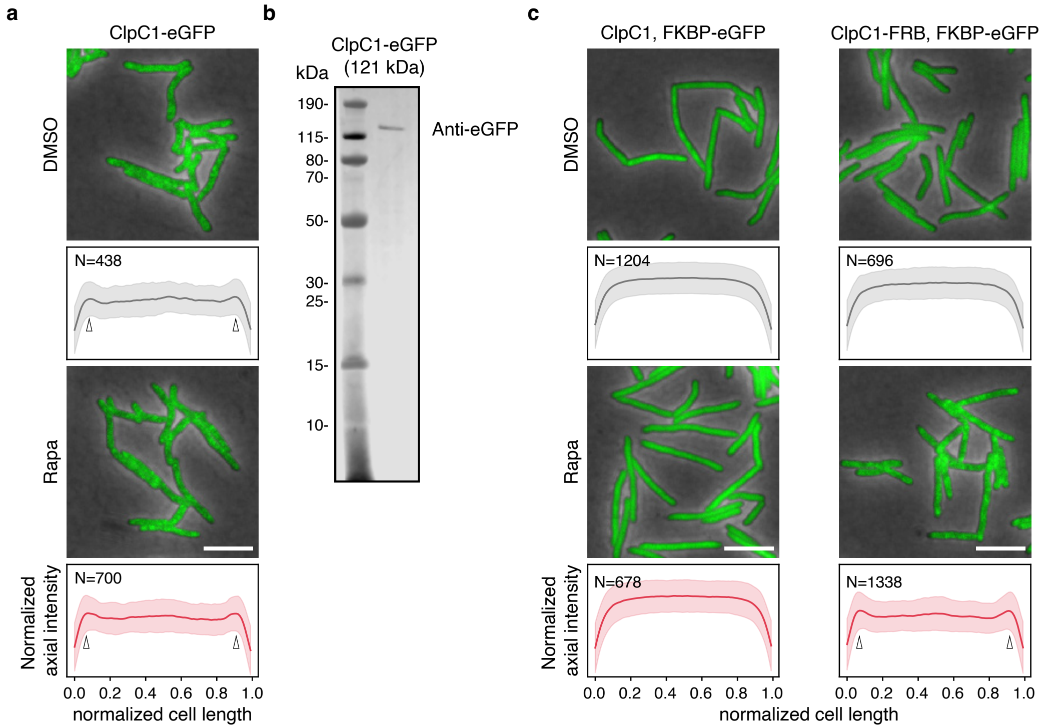

Supplementary Figure 4 I Rapamycin re-localizes FKBP-eGFP.
a Live cell, wide-field fluorescence microscopy images of cells expressing ClpC1 tagged at its chromosomal locus with eGFP and treated with DMSO (top) or 0.1 µg·mL^{-1} rapamycin (bottom). Scale bar, 5 µm. **b** Western blot analysis of ClpC1-eGFP. **c** Live cell, wide-field fluorescence microscopy images of cells expressing FKBP-eGFP in the WT *clpC1* (left) or *clpC1-frb* (right) background and treated with DMSO (top) or 0.1 µg·mL^{-1} rapamycin (bottom). For **a** and **c**, data are representative images selected from among 4 fields for each and are representative of two independent experiments. Normalized axial intensity of FITC signal across the cell widths of cells (N shown in each panel). Arrows highlight signal increases at the cell edges. Related to Fig. 2. Source data are provided with this paper.

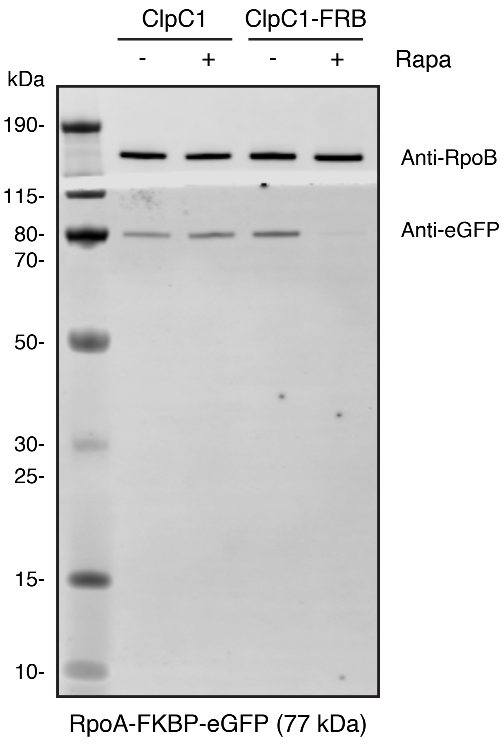

Supplementary Figure 5 | Uncropped western blot from Fig. 2e.
Western blot analysis of RpoA-FKBP-eGFP with DMSO or 0.1 µg·mL^{-1} rapamycin addition in the WT *clpC1* or *clpC1-frb* background. Density matched log phase cells incubated with DMSO or 0.1 µg·mL^{-1} rapamycin with shaking at 37°C for 24 h. Related to Fig. 2.

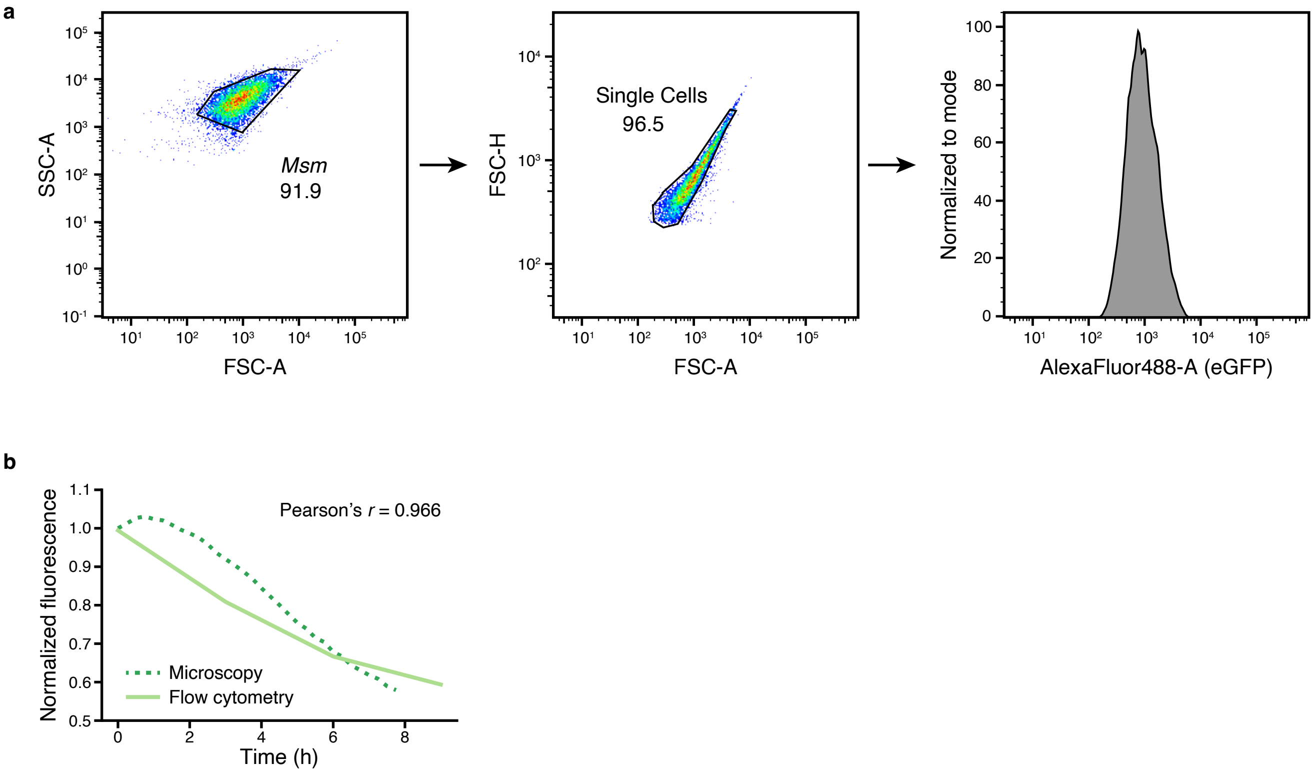

Supplementary Figure 6 | Flow gating strategy and RpoA degradation kinetics correlation plot.
a Representative flow gating strategy employed in this work. Gated single cells were used to determine fluorescence for data in Fig. 3c. **b** Correlation plot comparing fluorescent signal loss kinetics of RpoA-FKBP-eGFP by time-lapse microscopy and flow cytometry. Time-lapse data represents the median fluorescent signal of all cells and flow data represents the mean fluorescent signal of two technical replicates; both are normalized to time = 0 h. Related to Fig. 3. Source data are provided with this paper.

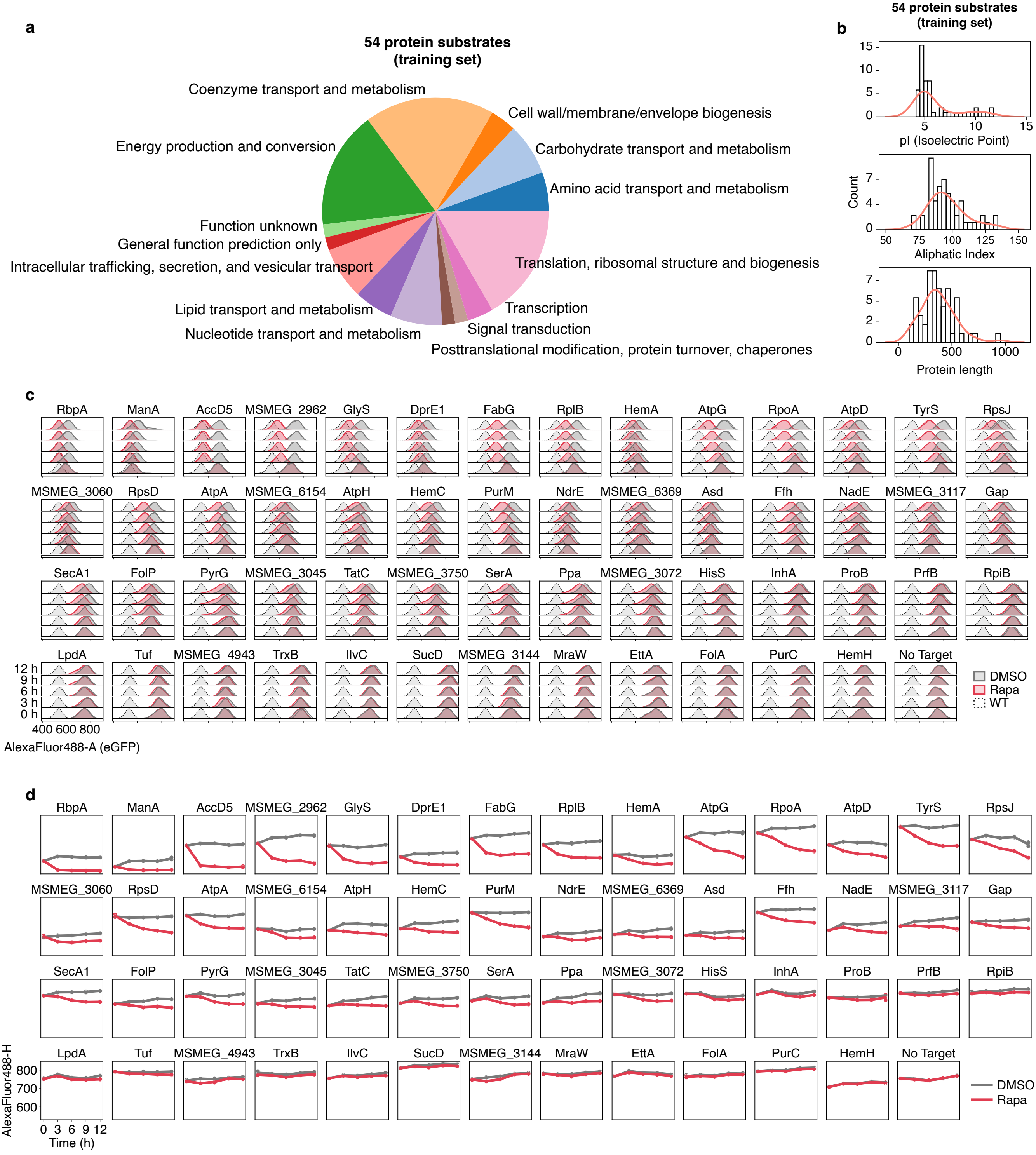

a
54 protein substrates (training set)
Coenzyme transport and metabolism
Cell wall/membrane/envelope biogenesis
Energy production and conversion
Carbohydrate transport and metabolism
Amino acid transport and metabolism
Function unknown
General function prediction only
Intracellular trafficking, secretion, and vesicular transport
Translation, ribosomal structure and biogenesis
Lipid transport and metabolism
Transcription
Nucleotide transport and metabolism
Signal transduction
Posttranslational modification, protein turnover, chaperones
b
54 protein substrates (training set)
pI (Isoelectric Point)
Count
Aliphatic Index
Protein length
c
RbpA ManA AccD5 MSMEG_2962 GlyS DprE1 FabG RplB HemA AtpG RpoA AtpD TyrS RpsJ
MSMEG_3060 RpsD AtpA MSMEG_6154 AtpH HemC PurM NdrE MSMEG_6369 Asd Ffh NadE MSMEG_3117 Gap
SecA1 FolP PyrG MSMEG_3045 TatC MSMEG_3750 SerA Ppa MSMEG_3072 HisS InhA ProB PrfB RpiB
LpdA Tuf MSMEG_4943 TrxB IlvC SucD MSMEG_3144 MraW EttA FolA PurC HemH No Target
12 h 9 h 6 h 3 h 0 h
400 600 800
AlexaFluor488-A (eGFP)
DMSO
Rapa
WT
d
RbpA ManA AccD5 MSMEG_2962 GlyS DprE1 FabG RplB HemA AtpG RpoA AtpD TyrS RpsJ
MSMEG_3060 RpsD AtpA MSMEG_6154 AtpH HemC PurM NdrE MSMEG_6369 Asd Ffh NadE MSMEG_3117 Gap
SecA1 FolP PyrG MSMEG_3045 TatC MSMEG_3750 SerA Ppa MSMEG_3072 HisS InhA ProB PrfB RpiB
LpdA Tuf MSMEG_4943 TrxB IlvC SucD MSMEG_3144 MraW EttA FolA PurC HemH No Target
AlexaFluor488-H
800 700 600
0 3 6 9 12
Time (h)
DMSO
Rapa

Supplementary Figure 7 | Mycobacterial proteins are differentially degraded with varying kinetics.
a Pie chart depicting the diversity of biological functions of the 54 tested substrates used for model training. Here the 54 proteins are grouped based on their COG (Clusters of Orthologous Genes) functional category annotations. **b** Histograms depicting the diverse protein characteristics of the 54 tested substrates. **c** Density plots depicting the flow cytometry fluorescence profiles of the 54 reporter strains incubated with DMSO or 0.1 $\mu g \cdot mL^{-1}$ rapamycin and sampled at the indicated times from two technical replicates. **d** The fluorescence median of flow data from (**c**) are calibrated by the median of the respective DMSO-treated, time-zero group, and transformed into normalized signal decay for all indicated targets. Related to Fig. 3. Source data are provided with this paper.

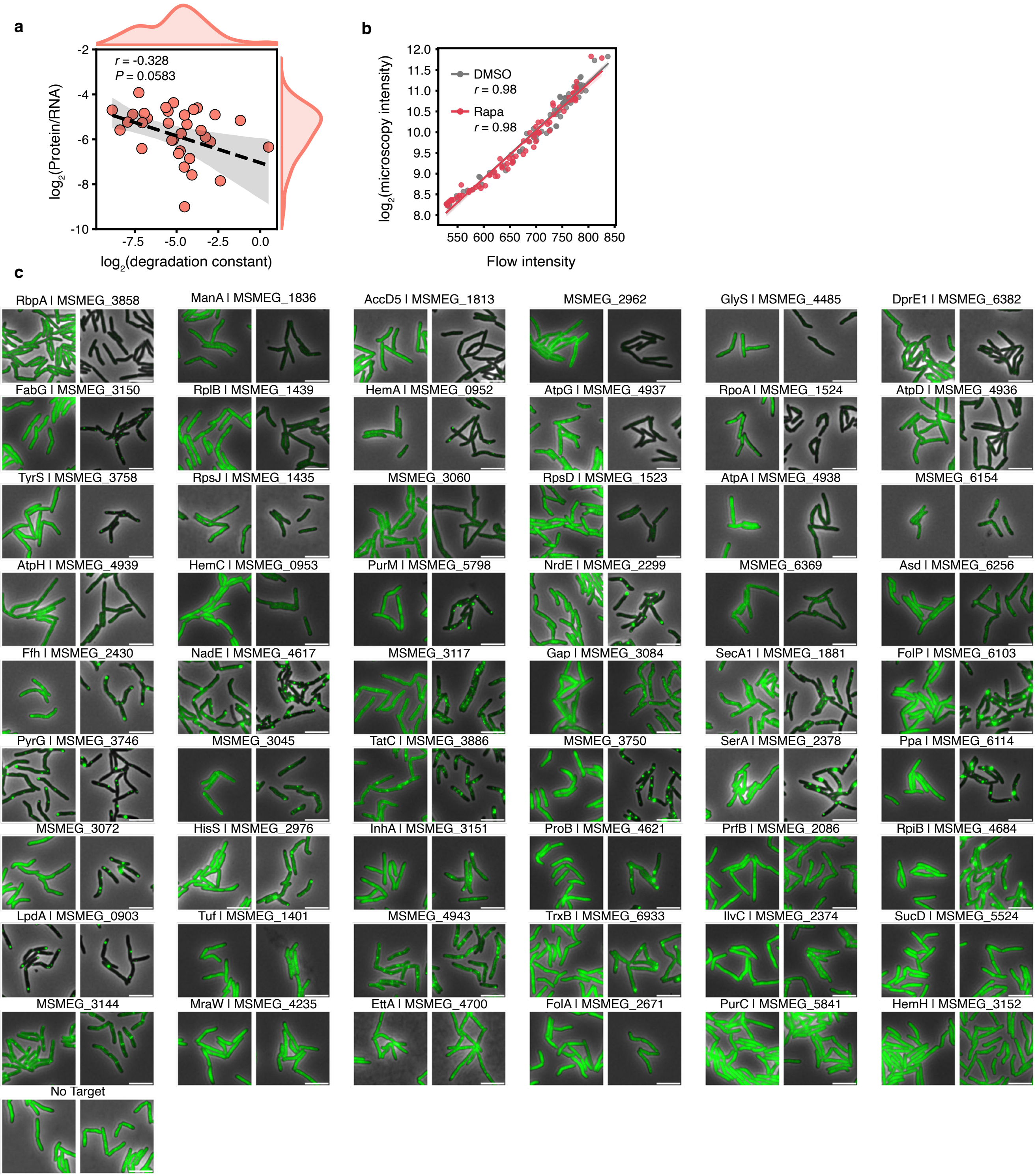

a
r = -0.328
P = 0.0583
log2(Protein/RNA)
log2(degradation constant)
-2
-4
-6
-8
-10
-7.5
-5.0
-2.5
0.0
b
DMSO
r = 0.98
Rapa
r = 0.98
log2(microscopy intensity)
Flow intensity
12.0
11.5
11.0
10.5
10.0
9.5
9.0
8.5
8.0
550
600
650
700
750
800
850
c
RbpA | MSMEG_3858
ManA | MSMEG_1836
AccD5 | MSMEG_1813
MSMEG_2962
GlyS | MSMEG_4485
DprE1 | MSMEG_6382
FabG | MSMEG_3150
RplB | MSMEG_1439
HemA | MSMEG_0952
AtpG | MSMEG_4937
RpoA | MSMEG_1524
AtpD | MSMEG_4936
TyrS | MSMEG_3758
RpsJ | MSMEG_1435
MSMEG_3060
RpsD | MSMEG_1523
AtpA | MSMEG_4938
MSMEG_6154
AtpH | MSMEG_4939
HemC | MSMEG_0953
PurM | MSMEG_5798
NrdE | MSMEG_2299
MSMEG_6369
Asd | MSMEG_6256
Ffh | MSMEG_2430
NadE | MSMEG_4617
MSMEG_3117
Gap | MSMEG_3084
SecA1 | MSMEG_1881
FolP | MSMEG_6103
PyrG | MSMEG_3746
MSMEG_3045
TatC | MSMEG_3886
MSMEG_3750
SerA | MSMEG_2378
Ppa | MSMEG_6114
MSMEG_3072
HisS | MSMEG_2976
InhA | MSMEG_3151
ProB | MSMEG_4621
PrfB | MSMEG_2086
RpiB | MSMEG_4684
LpdA | MSMEG_0903
Tuf | MSMEG_1401
MSMEG_4943
TrxB | MSMEG_6933
IlvC | MSMEG_2374
SucD | MSMEG_5524
MSMEG_3144
MraW | MSMEG_4235
EttA | MSMEG_4700
FolA | MSMEG_2671
PurC | MSMEG_5841
HemH | MSMEG_3152
No Target

Supplementary Figure 8 | Mycobacterial proteins are differentially degraded and sometimes re-localized with rapamycin.
a Association between the measured degradation potential ($\log_2$(degradation constant)) of *Msm* proteins and the steady-state protein-to-RNA ratio of their *Mtb* homologs. The absolute protein abundance of *Mtb* proteins, as measured by Schubert *et al.*[31], and their corresponding RNA expression profiles, as represented by RPKM and assayed by Płociński *et al.*[30], are $\log_2$-transformed and used as independent estimates of the steady-state protein and RNA abundance, respectively. *r* and *P* represent the coefficient and the *P* value of a two-sided Pearson's correlation test between measured degradation constants of *Msm* proteins and the steady-state protein-to-RNA ratios of their *Mtb* homologs. Note that only 34 of the 54 selected *Msm* substrates had their *Mtb* homologs present in both studies and included in this analysis. **b** Correlation plot comparing fluorescent signal intensity for each of the 54 tested targets by microscopy and flow cytometry. Samples stratified by treatment with DMSO or 0.1 µg·mL-1 rapamycin. **c** Live cell, wide-field fluorescence microscopy images of cells expressing selected target proteins with DMSO or 0.1 µg·mL^{-1} rapamycin and examined at 9 hours after treatment (extension of Fig. 3f), Scale bar, 5 µm. In **b**, Data are bounded by the 95% confidence interval. For **b**, microscopy data represents the median fluorescent signal of all cells and flow data represents the mean fluorescent signal of two technical replicates. In **c**, data are representative images selected from among at least 9 fields for each condition. Related to Fig. 3. Source data are provided with this paper.

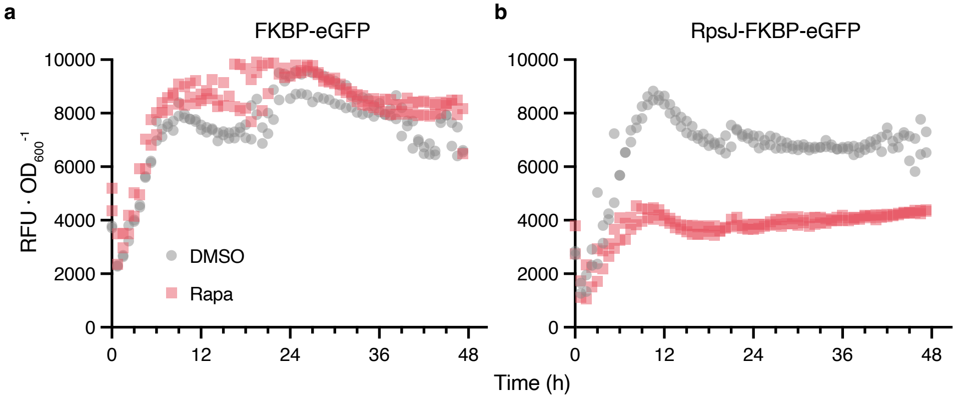

Supplementary Figure 9 l Rapamycin stably directs degradation of RpsJ for 48 h.

a-b Cell density (OD_{600})-normalized fluorescence of live cells as a proxy for protein levels of FKBP-eGFP (**a**) or RpsJ-FKBP-eGFP (**b**) in the *clpC1-frb* background. Density matched log phase cells incubated with DMSO or 1 $\mu g \cdot mL^{-1}$ rapamycin with shaking at 37°C. RFU = relative fluorescence units. Data are individually plotted technical replicate measurements. Related to Fig. 3. Source data are provided with this paper.

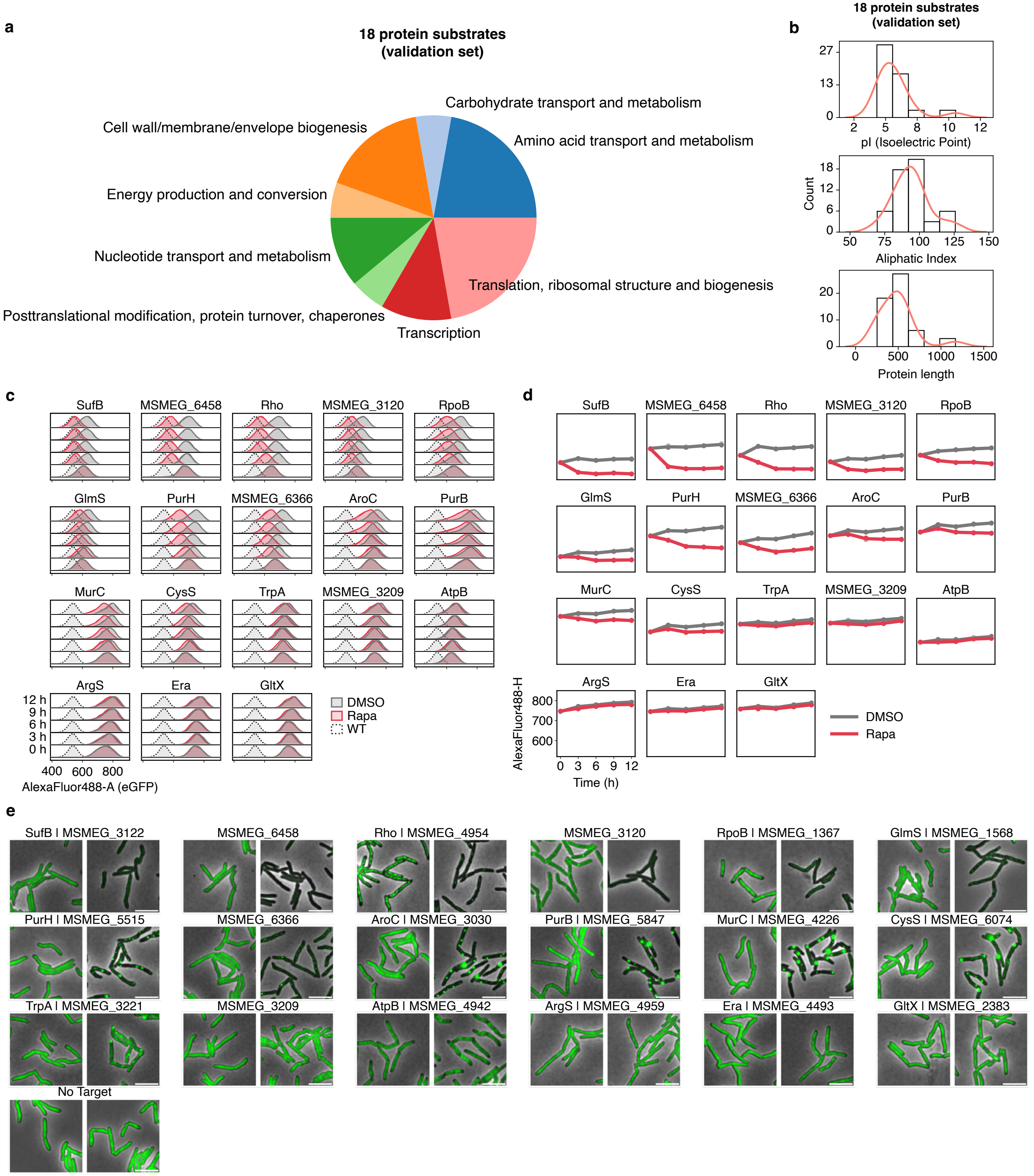
a
18 protein substrates (validation set)
Carbohydrate transport and metabolism
Cell wall/membrane/envelope biogenesis
Amino acid transport and metabolism
Energy production and conversion
Nucleotide transport and metabolism
Translation, ribosomal structure and biogenesis
Posttranslational modification, protein turnover, chaperones
Transcription
b
18 protein substrates (validation set)
pI (Isoelectric Point)
Aliphatic Index
Protein length
Count
c
SufB
MSMEG_6458
Rho
MSMEG_3120
RpoB
GlmS
PurH
MSMEG_6366
AroC
PurB
MurC
CysS
TrpA
MSMEG_3209
AtpB
ArgS
Era
GltX
12 h
9 h
6 h
3 h
0 h
DMSO
Rapa
WT
AlexaFluor488-A (eGFP)
d
AlexaFluor488-H
Time (h)
DMSO
Rapa
e
SufB I MSMEG_3122
MSMEG_6458
Rho I MSMEG_4954
MSMEG_3120
RpoB I MSMEG_1367
GlmS I MSMEG_1568
PurH I MSMEG_5515
MSMEG_6366
AroC I MSMEG_3030
PurB I MSMEG_5847
MurC I MSMEG_4226
CysS I MSMEG_6074
TrpA I MSMEG_3221
MSMEG_3209
AtpB I MSMEG_4942
ArgS I MSMEG_4959
Era I MSMEG_4493
GltX I MSMEG_2383
No Target

Supplementary Figure 10 | Function and feature diversity of the 18 protein substrates used for model validation.
a Pie chart depicting the diversity of biological functions of the 18 substrates used for model validation grouped by their COG functional category annotations. **b** Histograms depicting the diverse protein characteristics of the 18 substrates. **c** Density plots depicting the flow cytometry fluorescence profiles of the 18 validation reporter strains incubated with DMSO or 0.1 $\mu g{\cdot}mL^{-1}$ rapamycin and sampled at the indicated times from two technical replicates. **d** The fluorescence median of flow data from (**c**) are calibrated by the median of the respective DMSO-treated, time-zero group, and transformed into normalized signal decay for all indicated targets. **e** Live cell, wide-field fluorescence microscopy images of cells expressing each of the 18 validation substrates with DMSO or 0.1 $\mu g{\cdot}mL^{-1}$ rapamycin and examined at 9 hours after treatment. In **e**, data are representative images selected from among at least 9 fields for each condition. Related to Fig. 4. Source data are provided with this paper.

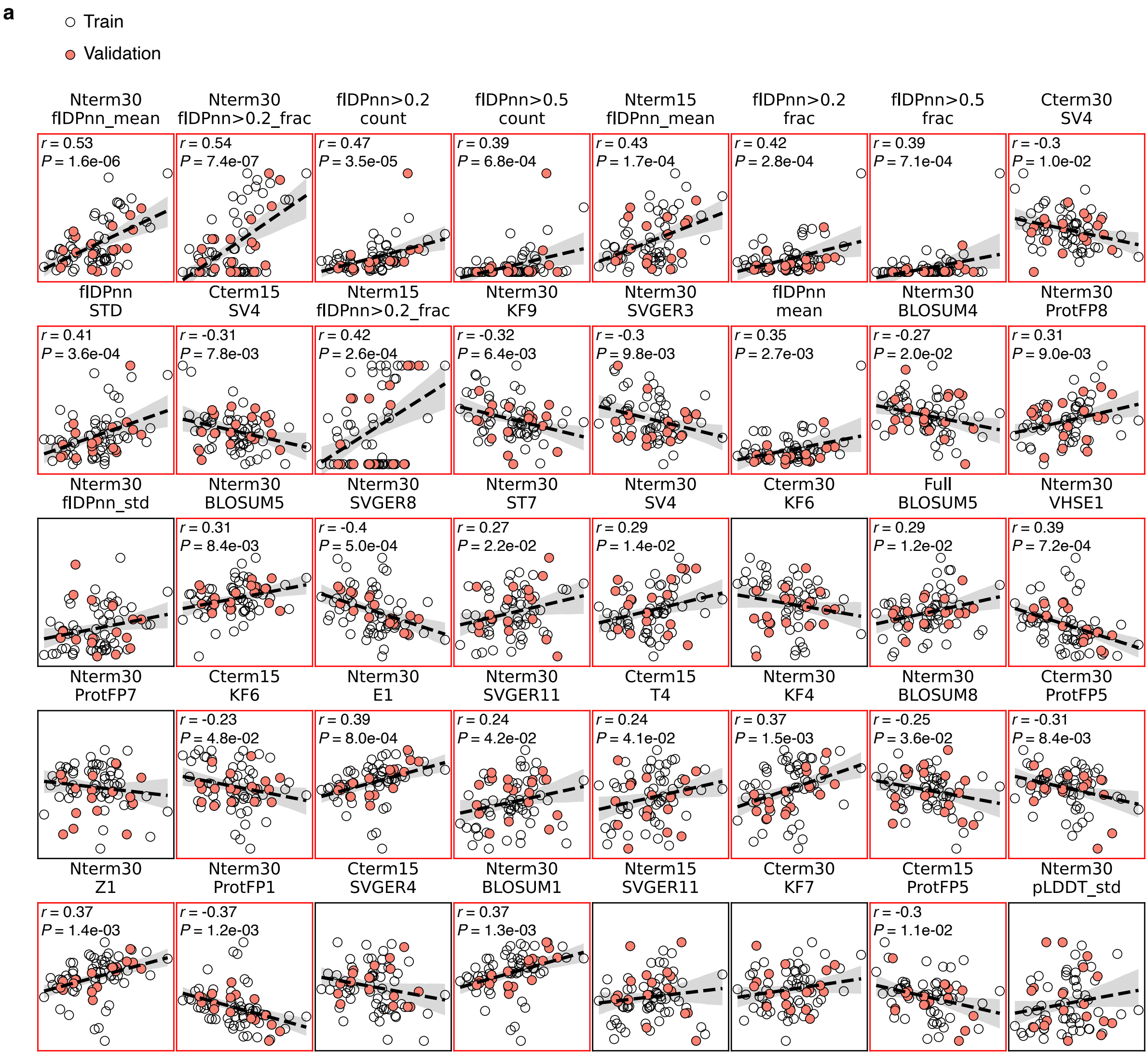

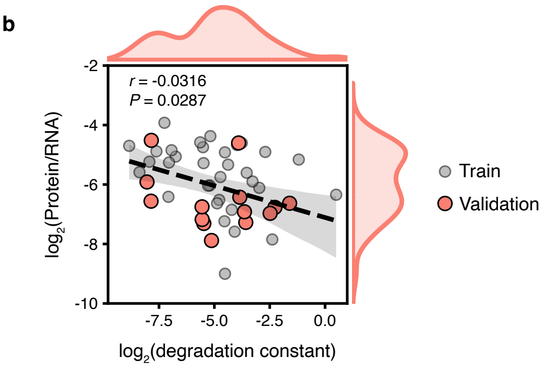

Supplementary Figure 11 l Visualization of linear association between selected protein features and measured degradation potential.
a Scatter plots demonstrating the degradation potential of the 72 tested substrates (54 for training and 18 for validation) across each of the 40 selected features, ordered by their relative contributions to model training (from left to right and from top to bottom). For features which exhibited statistically-significant linear associations with log_2-transformed degradation constant, the corresponding scatter plots are framed with red rectangles and provided with their Pearson's correlation coefficients *r* and *P* values. **b** Association between the measured degradation potential (log_2(degradation constant)) of 48 tested *Msm* proteins and the steady-state protein-to-RNA ratio of their *Mtb* homologs. The 48 *Msm* proteins encompass 34 from the training set and 14 from the validation set. Data in **a** and **b** are bounded by the 95% confidence interval. Related to Fig. 4. Source data are provided with this paper.

a

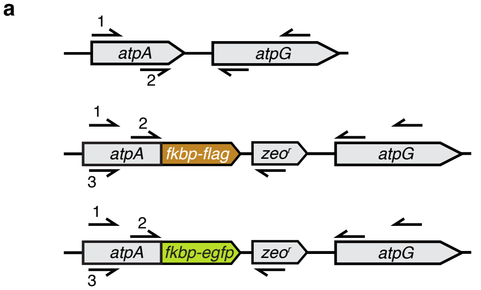

Primer pair	mc²155	*atpA::fkbp-flag*	*atpA::fkbp-egfp*
1	1.8 kb	2.8 kb	3.5 kb
2	0.3 kb	1.3 kb	2.0 kb
3	no band	1.4 kb	2.4 kb

b

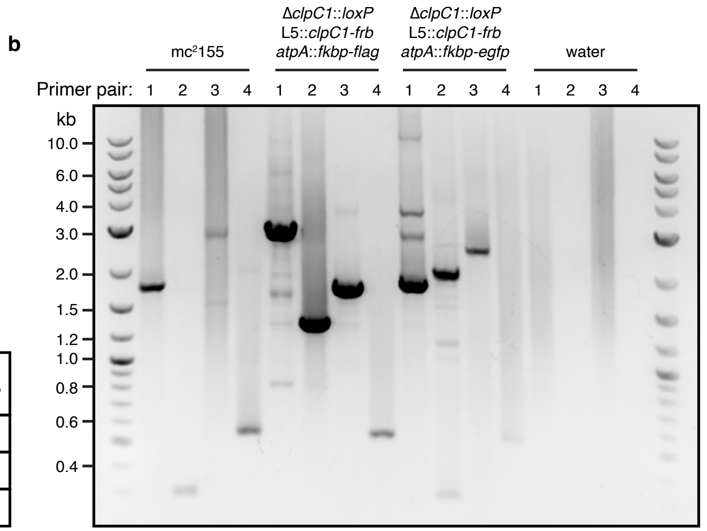

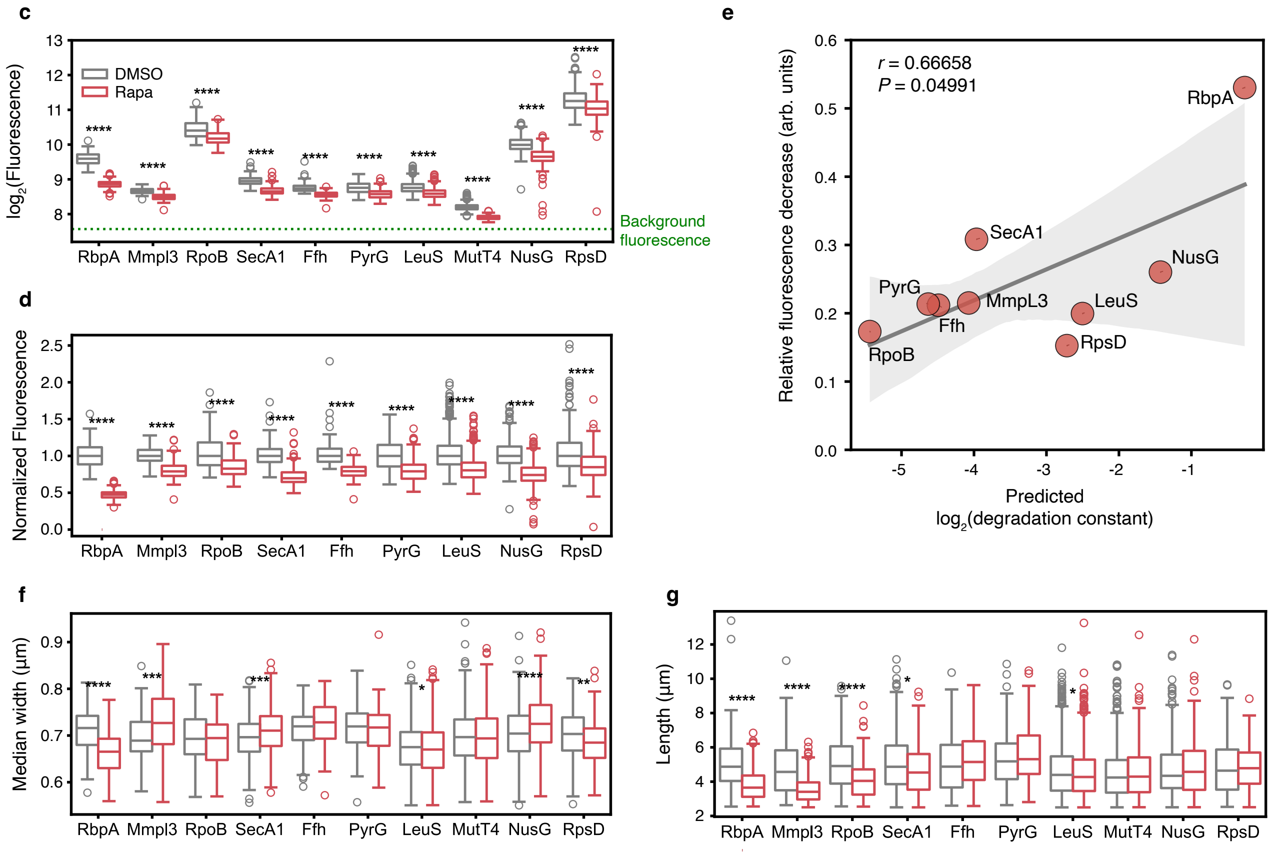

Supplementary Figure 12 | Phenotypic profiling of *Msm* strains subject to targeted degradation of endogenous proteins.
a-b PCR validation of the presence of a cryptic *atpA* locus duplication in a subset of the endogenously tagged strains. **a** Schematic depicting the design of validation PCR primers and their expected amplicon sizes. **b** Gel electrophoresis for the validation PCRs. Note that for primer pair 1, the *atpA-fkbp-egfp*, but not the *atpA-fkbp-flag* strain, yielded two PCR bands at ~1.8 kb and ~3.5 kb that corresponded to the co-presence of a WT copy and a tagged recombinant copy, respectively. **c** Boxplot displaying the $\log_2$-transformed cellular fluorescence profiles of the 10 endogenously tagged *Msm* strains. These strains were incubated with DMSO or 0.1 $\mu g \cdot mL^{-1}$ rapamycin and sampled after 9 hours for microscopy imaging. Background fluorescence was estimated by averaging the signal of imaging fields with no bacterial cells and marked by dashed green line. **d** Boxplot displaying the cellular fluorescence normalized to the populational fluorescence mean of the DMSO-treated control group. Background fluorescence as defined in (**c**) was subtracted before computing the normalized fluorescence profiles. MutT4 was excluded from this analysis as its steady state fluorescence was already close to the background, which compromised the interpretability of the radiometric normalization analysis. **e** Correlation analysis between machine-learning-predicted degradability ($\log_2$-transformed) and measured decline of relative fluorescence after 9 hours of rapamycin induction. **f-g** Profiles of cell width (**f**) and cell length (**g**) for the 10 chromosomally tagged *Msm* strains treated with DMSO or 0.1 $\mu g \cdot mL^{-1}$ rapamycin. The non-parametric Mann-Whitney U test was used to evaluate morphological differences between the two treatment groups. *, **, ***, and **** corresponds to a *P* value lower than 0.05, 0.01,0.001, and 0.0001, respectively. Data in **e** are bounded by the 95% confidence interval. For **c**, **d**, **f**, and **g**, statistical comparisons were made using measurements of at least 70 cells for each strain. The lower bound, centerline, and upper bound of the boxes represent the 1st (25th percentile), 2nd (median), and the 3rd quartile (75th percentile) of the dataset, respectively. The bottom and top whiskers represent values that deviate from the 1st and 3rd quartile by 1.5 times the interquartile range. Flier dots represent outliers. Related to Fig. 5. Source data are provided with this paper.

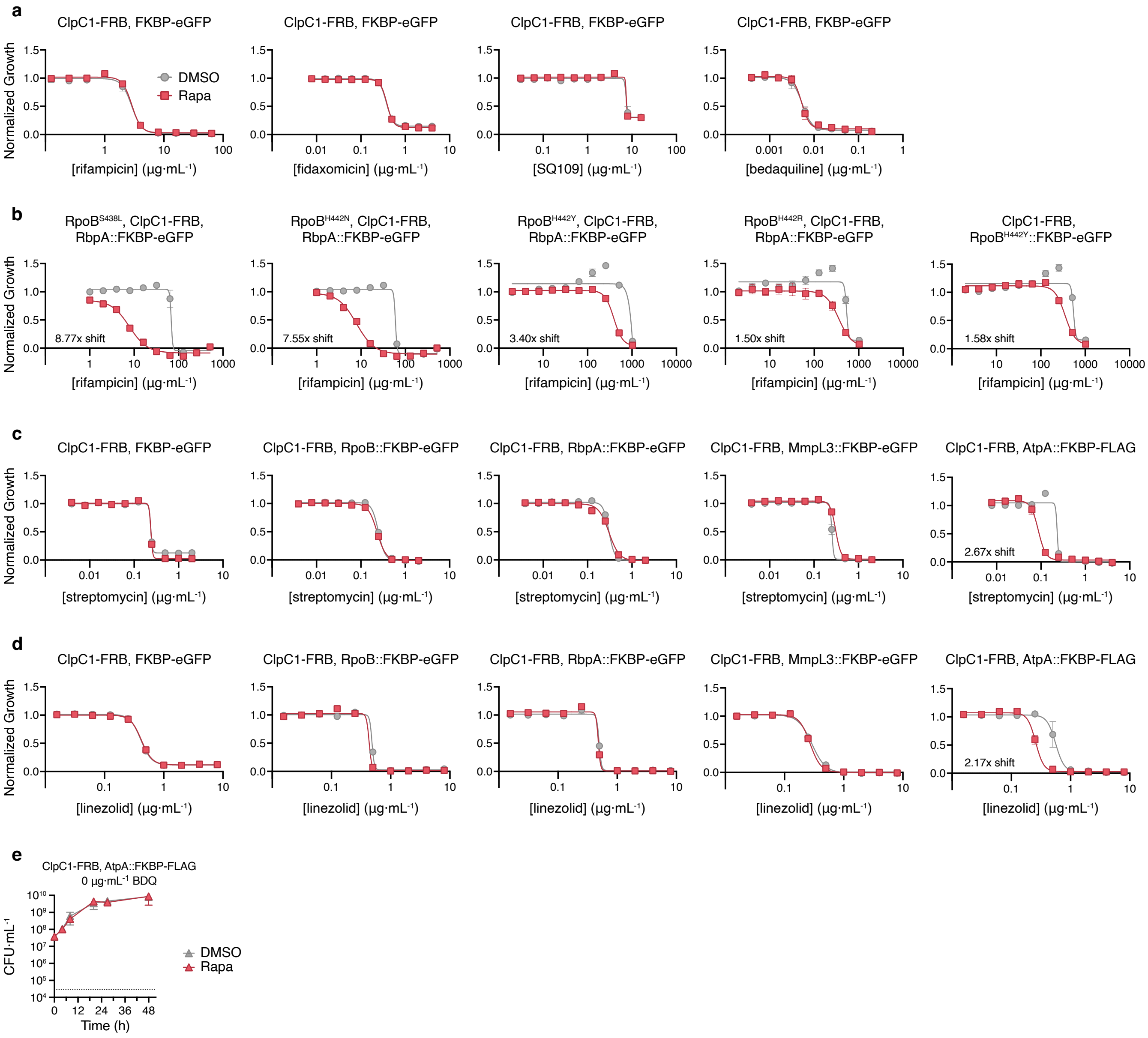

Supplementary Figure 13 | *Msm* dose response to antibiotics with targeted protein degradation.
a-d Half-maximal minimum inhibitory concentration (MIC_{50}) dose response measuring the sensitivity of indicated strains to rifampicin (**a-b**), fidaxomicin (**a**), SQ109 (**a**), bedaquiline (**a**), streptomycin (**c**), or linezolid (**d**) in media supplemented with DMSO or 0.1 µg·mL^{-1} or 0.5 µg·mL^{-1} rapamycin for all *atpA*::*fkbp-flag* tagged strains. Observed fold-shifts are denoted on the corresponding plot. **e** Growth curves measuring the number of individual colony forming units (CFU) of strains expressing AtpA-FKBP-FLAG when supplemented with either DMSO or 0.5 µg·mL^{-1} rapamycin. For **a-e**, data are mean ± s.d. of three technical replicates. Related to Fig. 5. Source data are provided with this paper.
