# Supplementary material for: Targeted protein degradation in mycobacteria uncovers antibacterial effects and potentiates antibiotic efficacy
Source: Nat Commun. 2024 May 14;15:4065. doi: 10.1038/s41467-024-48506-8 (PMC11094019; doi:10.1038/s41467-024-48506-8)
Supplement: Supplementary file 3 — Description of Additional Supplementary Files [file 41467_2024_48506_MOESM3_ESM.pdf]

File name: Supplementary Data File 1
Description: Complete list of sequence and structural features of the 348 candidate proteins.

File name: Supplementary Data File 2
Description: Complete list of sequence and structural features of the proteins used for model training and validation.

File name: Supplementary Data File 3
Description: Predicted degradation potential, gene essentially, and vulnerability of conserved Msm and Mtb proteins.

File name: Supplementary Data File 4
Description: Plasmids generated in this study.

File name: Supplementary Data File 5
Description: Strains generated in this study.

File name: Supplementary Data File 6
Description: Primers used in this study.
